# Comparisons of Fatty Acid Taste Detection Thresholds in People Who Are Lean vs. Overweight or Obese: A Systematic Review and Meta-Analysis

**DOI:** 10.1371/journal.pone.0169583

**Published:** 2017-01-06

**Authors:** Robin M. Tucker, Kathryn A. Kaiser, Mariel A. Parman, Brandon J. George, David B. Allison, Richard D. Mattes

**Affiliations:** 1 Department of Food Science and Human Nutrition, Michigan State University, East Lansing, Michigan, United States of America; 2 Office of Energetics, School of Health Professions, University of Alabama at Birmingham, Birmingham, Alabama, United States of America; 3 Nutrition Obesity Research Center, University of Alabama at Birmingham, Birmingham, Alabama, United States of America; 4 Department of Nutrition Science, Purdue University, West Lafayette, Indiana, United States of America; Universitat Potsdam, GERMANY

## Abstract

Given the increasing evidence that supports the ability of humans to taste non-esterified fatty acids (NEFA), recent studies have sought to determine if relationships exist between oral sensitivity to NEFA (measured as thresholds), food intake and obesity. Published findings suggest there is either no association or an inverse association. A systematic review and meta-analysis was conducted to determine if differences in fatty acid taste sensitivity or intensity ratings exist between individuals who are lean or obese. A total of 7 studies that reported measurement of taste sensations to non-esterified fatty acids by psychophysical methods (e.g.,studies using model systems rather than foods, detection thresholds as measured by a 3-alternative forced choice ascending methodology were included in the meta-analysis. Two other studies that measured intensity ratings to graded suprathreshold NEFA concentrations were evaluated qualitatively. No significant differences in fatty acid taste thresholds or intensity were observed. Thus, differences in fatty acid taste sensitivity do not appear to precede or result from obesity.

## Introduction

Flavor is an important determinant of both food selection [1,2] and rejection [3]. Though agreement is not uniform, a widespread view is that the flavor of food reflects the summation of all of its sensory properties [4]. The relative contribution of each sensory property varies with the food system and concentration of the contributing stimuli. Stimuli that are rated as unpleasant when sampled alone (e.g., a bitter compound in water) may make an important positive contrition to the flavor profile of a food when present at an appropriate low concentration. For example, bitterness adds to the appeal of popular items such as chocolate, coffee, and wine. Fatty acids may resemble bitter compounds in this regard.

Evidence now strongly indicates that humans can detect non-esterified, long chain fatty acids (NEFA) in the oral cavity, referred to as fat taste or, more recently, “oleogustus” [5]). This evidence includes a growing body of human psychophysical work, e.g. [6–17] as well as identification of receptors in human lingual epithelium [18,19] and transduction mechanisms [20]).

Given that fats are especially energy dense, special attention has been given to their role in promoting weight gain and obesity, but evidence is mixed [21,22]. Although the majority of fat in the diet is in the form of esterified fatty acids (triacylglycerols), NEFA exist in fat-containing foods while salivary NEFA concentrations increase to levels that are detectable by many individuals when these foods are masticated [23,24]. When NEFA are present alone and in high concentrations, they are generally rated as unpleasant [6]. However, at peri-threshold concentrations and in selected contexts NEFA might be rated favorably. Like umami, the sensory quality of NEFA is difficult to describe, but recent work indicates it is a unique sensation [5]. With taste playing an important role in food selection and the considerable inter-individual variation in oleogustus sensitivity that has been previously documented, e.g., 2–4 orders of magnitude [13,25], explorations of the relationships between adiposity, food intake, and taste sensitivity to NEFA [6–17] have occurred.

Studies that have examined relationships between taste sensitivity to NEFA and adiposity report varied findings. When associations between taste sensitivity to NEFA and weight status are found, they are typically negative correlations; that is, as body weight increases, sensitivity decreases [8,9,11–13,15,26]. Several explanations have been put forth to explain these findings. First, as NEFA at higher concentrations impart an aversive taste quality, a reduced ability to detect NEFA could result in lower rejection likelihood and greater intake [16]. Given that NEFA are more energy dense than some other food components, this increased consumption could lead to elevated body weight. Such an explanation may account for a discrepancy in intake for those who are sensitive versus less sensitive but would not explain a level of intake that exceeds energy needs in those with obesity. Alternatively, it has been suggested that a high-fat diet leads to habituation and the need for an increased stimulus to generate a desired positive oral response [13–15], leading to increased intake and body weight. This explanation is predicated on the taste being hedonically pleasant; whereas, all current evidence supports the opposite. Additionally, decreased sensitivity could result in decreased satiety [27], leading to increased intake and weight gain. Here, a causal association between taste sensitivity and satiety is required, but evidence for such a mechanism is lacking.

Other studies find no association between taste sensitivity to NEFA and adiposity [6,7,10,14,16,17]. The absence of a relationship between taste sensitivity and body weight is in agreement with work that has examined relationships between other prototypical primary taste qualities and body mass index (BMI) [28–33]. It should also be noted that sensitivity is not always associated with liking or preference for stimuli present at the threshold or suprathreshold concentrations we typically experience [34–38], so a lack of association with sensitivity and body weight is not necessarily surprising. The sensory contribution of one food ingredient may easily be masked by the myriad of other chemicals in foods, and sensory properties are only one of many determinants of food choice.

Nevertheless, given the considerable potential therapeutic implications of identifying and modulating a causal relationship between taste sensitivity to NEFA and weight status, we conducted a systematic review and meta-analysis that aimed to address the question: Do individuals who are overweight or obese differ from lean individuals in threshold sensitivity and suprathreshold intensity scaling of NEFA in model stimuli? We relied on model stimuli as these minimize confounding factors that arise when fatty acids are incorporated into complex food matricies. It is acknowledged that the ecological implications of findings from model systems is uncertain; however, the intent here is to determine whether lean and obese differ in sensitivity to non-esterified fatty acids, and model systems provide the most sensitive test of this question. Perceived differences in foods may relate more to discrimination between competing sensory properties, a different question. We operationalized the research question by reviewing studies that performed taste threshold tests or taste intensity scaling tests and compared the group means among participants of different weight categories.

## Materials and Methods

The review was registered with PROSPERO, registry # CRD42015024187, prior to data collection (http://www.crd.york.ac.uk/PROSPERO/).

### Data Sources

Reports of studies were retrieved using searches performed in the following electronic databases: English language articles in PubMed, SCOPUS, ProQuest PsycInfo, and Dissertation Abstracts. Data were extracted from the published reports of the final selected studies with two exceptions (noted later) for the purposes of description and meta-analysis.

### Study Search Terms

We used the following search string in PubMed (shown as an example): (((((taste) OR "taste perception") OR "taste threshold") AND Humans[Mesh])) AND ((((((((((((((((((dietary fat*) OR fatty acid*) OR lauric) OR myristic) OR myristoleic) OR palmitic) OR stearic) OR vaccenic) OR oleic) OR linoleic) OR stearidonic) OR gondoic) OR arachid*) OR behenic) OR *pentaenoic) OR erucic) OR *hexanoic Filters: Humans) AND Humans[Mesh]). Additionally, we searched for “fat discrimination” and “fat taste” in the index of the Flavor and Fragrance Journal.

### Inclusion Criteria

1. Studies of human subjects of any developmental age published prior to November 1, 2015 were eligible for inclusion.
2. Participants were comprised of at least two groups, one group consisting of persons with a mean BMI > = 25, with the remainder in the comparator group having a mean BMI between 18 and 25, inclusive. For this study, a BMI of 25.0–29.9 was considered overweight; a BMI of ≥ 30.0 was considered obese.
3. Studies that report measurement of taste sensitivity to non-esterified fatty acids by psychophysical methods (e.g., detection thresholds as measured by a 3-alternative forced choice ascending methodology; intensity as measured by intensity scaling) were included.
4. There must be at least one portion of the protocol that involves use of nose clips, or another apparatus to prevent nasal exposure to the test solutions to insure taste, not olfaction, is being measured.
5. Non-esterified fatty acids are used to test taste thresholds/intensity and must be one of the following:
  - Lauric 12:0
  - Myristic 14:0
  - Myristoleic 14:1
  - Palmitic 16:0
  - Palmitoleic 16:1
  - Stearic 18:0
  - Vaccenic 18:1
  - Oleic 18:1
  - Linoleic 18:2
  - Alpha linolenic 18:3
  - Stearidonic 18:4
  - Gondoic 20:1
  - Arachidic 20:0
  - Arachidonic 20:4
  - Behenic 22:0
  - n-3 Eicosapentaenoic 20:5
  - Erucic 22:1
  - n-3 Docosapentaenoic 22:5
  - n-3 Docosahexaenoic 22:6

### Exclusion Criteria

1. Studies that lack taste sensitivity measures for specific fatty acid concentrations (i.e., aggregated data such as detection below a cut point, were not included)
2. Studies that utilize triglycerides or foods with uncharacterized NEFA profiles as taste stimuli
3. Studies that tested non-esterified fatty acids with 12 or fewer or more than 22 carbons, or any oxidized form of fatty acids
4. Studies that do not provide adiposity or BMI status of participants
5. Studies where participants have a condition or medical history of a condition known to cause taste discrimination disturbances (cancer chemotherapy treatment, head or brain cancers, current smokers)
6. Studies on pregnant women
7. Studies employing trained taste panelists

### Filtering Steps

We combined all database search results into a master reference database, deleted any duplicate references and set aside review articles. Two authors (KAK and MAP) independently reviewed the initial list and compared selections, evaluating all against the pre-defined criteria. If the abstract did not contain enough information to clearly exclude a paper, we examined the full paper per inclusion and exclusion criteria. We identified other studies based on the expert knowledge of the field by the first and senior authors (RMT and RDM) and by searching references in the methods sections that cited other papers as original reports. Finally, we reviewed reference lists of pertinent reviews and hand searched the index of all articles in the *Flavor and Fragrance Journal*. Two authors (KAK and BJG) independently extracted the data related to sample size and outcomes and wrote authors to request missing data or to provide clarifying information.

### Statistical Analysis

We used Review Manager [39] and Microsoft^®^ Excel to calculate the standardized mean differences between treatment and control groups when sample sizes, means and SEs/SDs were reported, or if exact p values were given between groups for change values within groups. These data were used to generate the forest plots for each comparison using Review Manager, version 5.3.5 [39].

### Risk of Bias Assessment

Two authors (KAK and MAP) independently reviewed each paper and evaluated the areas of potential risk of bias according to the Cochrane Handbook guidelines [40]. Discrepancies in ratings were discussed until consensus was reached. The risk of bias summary figures were generated with the Review Manager software, version 5.3.5 [39]. We used the following categories for assessment due to the special nature of the testing methods and study design: stimuli presented in randomized order, participants visually blinded to stimuli, participants blocked from olfactory input (nose clips), incomplete outcome data (attrition bias or procedural failure), researchers blinded to solution concentrations, statisticians blinded to group categories (detection bias), selective reporting (reporting bias), other potential sources of bias.

## Results

**Fig 1:**
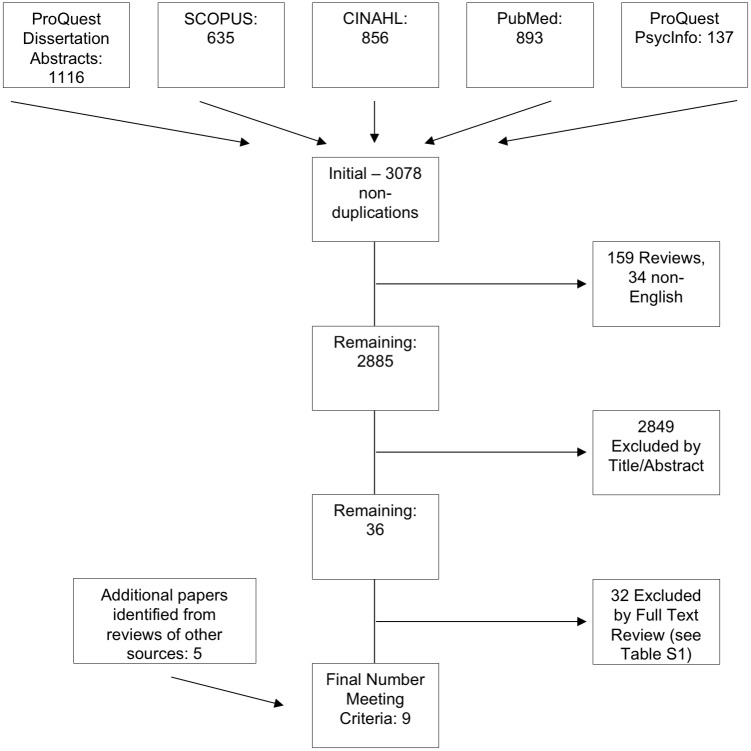
PRISMA flow diagram of the literature search selection process.

See Fig 1 for the literature search and study selection flow diagram. A total of 9 studies met our inclusion and exclusion criteria [6–8,10,12,14,16,17,26]. Table 1 contains a list of descriptions about these studies. S1 Table contains a list of the studies that were excluded and reasons for exclusion. The most common reasons for studies to be excluded were that there were no reported comparisons among weight categories or the stimuli solutions (e.g., raw milk based, puddings) used were of a complex nature and provided no way to determine the effective sensory stimulus for the specific fatty acids in question. Because the original way in which the data were reported in [10,17], we obtained the raw data to perform the calculations reported herein. Other notes about data requests are in Table 1. The study reported in [12] met our inclusion criteria, but the reported group comparison statistics were highly implausible (F-value of 51,000, or possibly 51.000 due to inconsistent decimal and comma use) and attempts to clarify the value were not successful. Additionally, it was not clear in this study that all participants used nose clips, which may have compromised the integrity of the detection method.

**Table 1:** Studies included in the meta-analysis.

**Study**	**Study Locale**	**Population Sample Tested**	**Study Design/ Detection Method**	**Fatty Acid(s) tested**	**Stimulus Matrix**	**Outcomes Among Weight Groups**
**Chevrot, 2014** [7]	France	Men, n = 59; Overnight fasted	3-AFC sip and spit procedure for threshold detection	Linoleic Acid— 18 solutions ranging from 0.00028%#x2014;5%	Mineral water mixed with acacia gum, paraffin oil, & EDTA	No difference: Obese (n = 29) threshold M = 1.007% wt:wt, SEM = 1.842; Lean (n = 30) M = 0.403% wt:wt, SEM = 0.789; p = 0.11.
**Daoudi, 2015** [8]	Algeria	Teenagers (46% female), n = 165; Overnight fasted	3-AFC sip and spit procedure for threshold detection	Oleic Acid– 8 solutions ranging from 0.018–12 mmol/L (diluent not specified)	0.01% acacia gum in stimulus and control	Higher threshold in obese: Obese (n = 83) threshold M = 2.57 mmol/L, SD = 0.28; Lean (n = 82) M = 1.33 mmol/L, SD = 0.15, p <0.0001.
**Mattes, 2009** [10] §	USA	Adults, n = 35 (62.3% female). Mean BMI = 24.5 kg/m^2^; no food drink or oral care products 2 hours prior to testing	2-AFC applied to tongue with sterile cotton swabs for threshold detection, rinsed with deionized water between samples; Intensity ratings were obtained using the general Labled Magnitude Scale (gLMS)	Stearic, Lauric, Caproic— 5 solutions ranging from 0.0028%—5% for threshold detection	Deionized water with EDTA, 5% gum acacia, 5% mineral oil; warmed to 67°- 69°C	No group comparison done in original report. Raw data obtained for weight group analysis of taste thresholds.
**Sayed, 2015** [12]	Algeria	Children, n = 116 (Mean age = 8 yrs, 49% girls, 49% obese); Overnight fasted	3-AFC sip and spit procedure for threshold detection; rinsed mouth between each set of samples	Oleic Acid– 8 solutions ranging from 0.018–12 mmol/L	Deionized water with EDTA and 0.01% (w/v) acacia gum	Higher threshold in obese: F(1,114) = 51,000; p < 0.000001 *(wrote author for raw means and SDs—this F value is highly implausible—no reply received)*.
**Stewart, 2011** [26]	Australia	Men, n = 19; Age range 19–58); no food drink or oral care products/ gum 1 hour prior to testing	3-AFC procedure for threshold detection	Oleic Acid– 12 solutions ranging from 0.02–12 mmol/L	“Long-life non-fat milk” with EDTA, 5% wt:vol gum acacia and liquid paraffin	Higher thresholds in overweight or obese compared to lean, respectively (geometric mean, SEM): 7.9, 0.1 mmol/L vs. 4.1, 0.4 mmol/L, p < 0.05 *(wrote author for raw means and SDs)*.
**Stewart, 2012** [14]	Australia	Adults, N = 31, Mean age 35.6 yrs; sex ratio not reported; no food drink or oral care products/gum 1 hour prior to testing	3-AFC procedure for threshold detection	Oleic Acid– 12 solutions ranging from 0.02–12 mmol/L	“Long-life non-fat milk” with EDTA, 5% wt:vol gum acacia and liquid paraffin served at room temperature	No difference in baseline testing between lean (n = 19) and OW/OB (n = 12) respectively (M, SD): before high fat diet phase: 2.9, 3.2 versus 4.6, 4.2; before low fat diet phase: 4.4, 4.2 versus 5.5, 4.7. *(wrote author for n per randomized group at baseline)*.
**Tucker, 2013** [6]	USA	Adults, n = 54, Mean age 25.6 yrs; 74% women	3-AFC procedure for threshold detection	Oleic Acid– 18 solutions ranging from 0.01 mM to 180 mM	Deionized water, 12% gum arabic and 0.01 xanthan gum with EDTA	No difference in baseline testing: Lean median step 3 (56.8 mM), SIQR = 5.5; Overweight median step 13 (0.18mM), SIQR = 6.
**Tucker, 2014** [16]	USA	Adults, n = 48, Mean age 28.5 yrs; 64.5% women	Modified staircase procedure: 3.2mM starting concentration with a 2- down, 1-up rule: thresholds determined from average of last 4 of 5 reversals	Oleic Acid– 5% w/v emulsion diluted to a range of 39 concentra-tions	Deionized water, 12% gum arabic and 0.01 xanthan gum with EDTA	Mixed models results show intercepts not different between lean vs. OW and lean vs. OB. Intercepts between OW and OB were different (p = .04).
**Tucker, 2015** [17] §	USA	Adults (n = 549) and children (n = 180);	Intensity rating on a 100 mm visual analog scale anchored with “extremely weak” and “extremely strong”. Test strips presented in random order and applied for 45 seconds; alternated tongue sides between strips	Linoleic Acid—one of three concentrations or a blank: 0.06%, 0.15%, or 0.38% v/v by calculation	Edible strips prepared with a solution of pullulan-hydroxy-propyl-methyl- cellulose (HPMC) combined with a stable emulsion of 0.5% w/v LA, 12% w/v gum Arabic, 0.01% w/v EDTA and 0.01% w/v TBHQ	Adults and children. Raw data summaries by weight categories provided by first author.

Abbreviations: OW—overweight; OB—obese; 3-AFC**—** 3-alternative forced choice ascending procedure; 2-AFC**—** 2-alternative forced choice ascending procedure; BMI—body mass index; EDTA—ethylenediaminetetraacetic acid; TBHQ—tert-Butylhydroquinone; SIQR—semi-interquartile range; SEM—standard error of the mean;

^§^—tested taste intensity ratings for solutions at the different concentrations.

### Meta-Analysis for Threshold Detection Among Weight Groups

**Fig 2:**
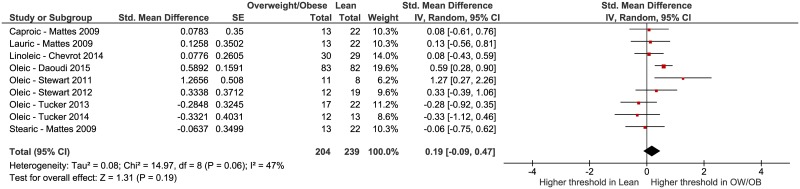
Forest plot of fatty acid taste thresholds of groups of participants who were lean versus overweight or obese from 7 included studies [6–8,10,14,16,26].

Five studies reported testing of oleic acid [6,8,14,16,26]; however, one study [7] evaluated linoleic acid detection and one [10] reported on caproic, lauric and stearic acid taste. The threshold concentration data from the selected studies were not consistently reported using the same units of measure, so we opted to calculate the standardized mean differences (SMDs) among the comparator weight groups. Fig 2 represents the SMDs for each included study (except for [12] due to previously mentioned concerns about the methods and data reported) and indicates that the cumulative effect estimate among the studies is not statistically significant: SMD = 0.19, 95% CI = -0.09 to 0.47, *p* = 0.19 overall. Moderate heterogeneity is present, I^2^ = 47%. If we included the Sayed 2015 [12] study using a more likely SMD based on an *F* value of 51 instead of what is reported (51,000), the overall estimate for all 7 studies is still not significantly different from 0 (SMD = 0.32, 95% CI = -0.05 to 0.69) and the heterogeneity increases markedly (I^2^ = 75%).

**Fig 3:**
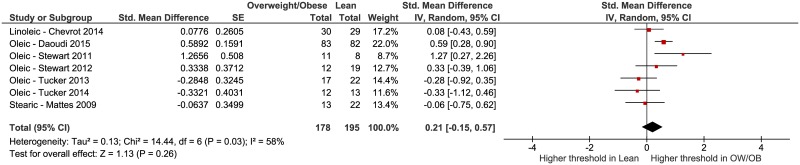
Forest plot of fatty acid taste thresholds of long-chain fatty acids of groups of participants who were lean versus overweight or obese [6–8,10,14,16,26].

We conducted additional analyses to determine if chain length or individual fatty acid influenced the results. First, we removed the data from the Mattes 2009 [10] study that tested caproic and lauric acid and re-ran the analysis (Fig 3). This was done because differences in sensitivity have been identified based on chain length [41]. Again, no significant effect was observed: SMD = 0.25, 95% CI = -0.15 to 0.65, *p* = 0.23 overall. Moderate to high heterogeneity is present, I^2^ = 62%. Second, we examined studies that only tested oleic acid [6,8,14,16,26], the SMD = 0.29, 95% CI = -0.21 to 0.79, *p* = 0.26 for overall effect. Moderate to high heterogeneity is present among these five studies, I^2^ = 67%.

### Intensity Ratings Among Weight Groups

Two of the included studies reported on the subjective rating of taste intensity of linoleic [17] and stearic, lauric, and caproic acids [10]. We have opted to describe the results qualitatively due to the limited number of studies and varied stimuli. The Tucker 2015 [17] study protocol asked participants to rate the intensity of four concentrations of linoleic acid, ranging from 0.0% w/v to 0.38% w/v using a 100 mm visual analog scale. Participants were classified as non-obese (N = 236) or obese (N = 304), based on body fat percentages of ≥25% in males and ≥30% in females rather than by BMI. No differences in fat taste intensity ratings were observed between non-obese and obese adults with the exception that non-obese participants rated the 0.15% w/v (“medium”) concentration as more intense (P = 0.03). The Mattes 2009 [10] study obtained both thresholds and intensity ratings for stearic, lauric, and caproic acid at specific tongue sites. Intensity was assessed using the general Labeled Magnitude Scale. Tested concentrations ranged from 0.00028% to 5% for each of the three fatty acids. No differences in thresholds or intensity ratings were observed among lean (N = 22), overweight (N = 8), and obese (N = 5) participants. Thus, the existing evidence does not indicate that BMI is significantly related to the ability to scale the intensity of oleogustus with stimulus concentration among the samples tested.

### Risk of Bias Assessment

**Fig 4:**
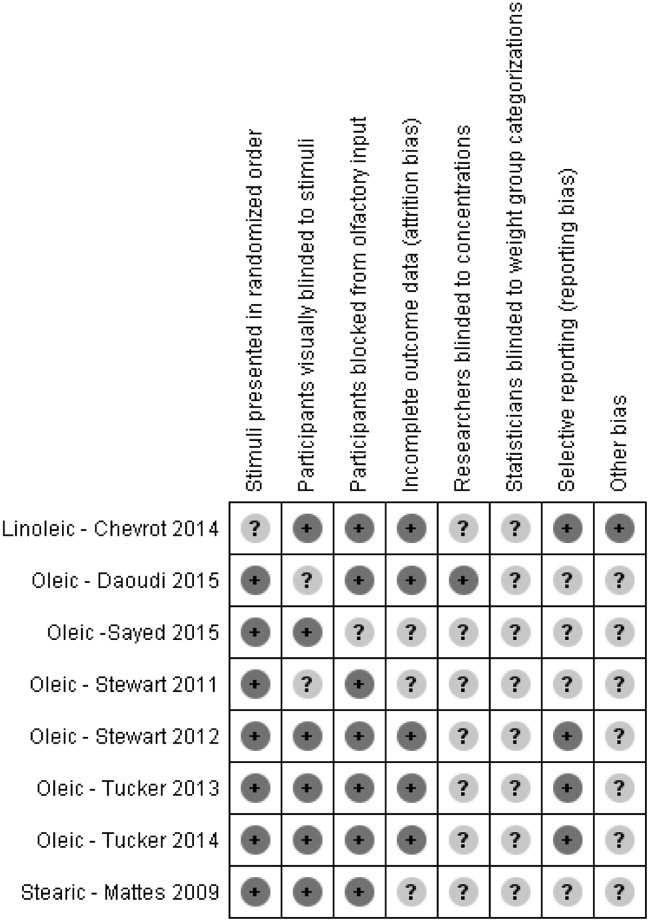
Risk of bias assessment summary table.

**Fig 5:**
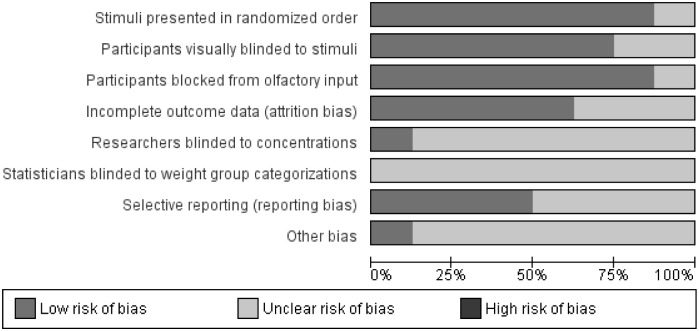
Risk of bias assessment graph.

Risk of bias assessment for the threshold studies (Figs 4 and 5) was performed independently by two authors (KAK and MAP) and items were discussed to reach agreement. We used the following categories for assessment due to the special nature of the testing methods and study design: stimuli presented in randomized order, participants visually blinded to stimuli, participants blocked from olfactory input (nose clips), incomplete outcome data (attrition bias or procedural failure), researchers blinded to solution concentrations, statisticians blinded to group categories (detection bias), selective reporting (reporting bias), other potential sources of bias.

While none of the information reported in the included studies indicated a high risk of bias, there was generally much unclear risk of bias due to a lack of explicit reporting. Not stating that statisticians were blinded to weight group categorizations was the most common indication for unclear risk of bias, with no studies reporting that statisticians were blinded. We found an overall low risk of bias related to participant influences: blocking participants’ olfactory input, randomizing the stimuli presentation order (when the method allowed) and visually blinding participants to the presented solutions. There was a moderate amount of unclear risk of attrition bias and selective reporting, and very few studies reported blinding researchers to solution concentrations (often difficult due to the methods employed).

## Discussion

Taste is the sensory system most closely aligned with nutrition with each of its component qualities reportedly tuned to different nutrients. Recent evidence that there is a taste component to fat complements this view. The questions that have emerged are whether individual differences in taste responses to NEFA could contribute to differential fat intake, body weight and fat mass, or alternatively, does obesity alter taste responses for NEFA?

Several mechanisms linking taste sensitivity to NEFA and adiposity have been proposed. Single nucleotide polymorphisms for the putative fatty acid receptor CD36 have been described and linked to altered taste thresholds [11,42] but not presently with BMI. Saliva can influence responses to fatty acids by multiple mechanisms [43]. Lower salivary flow rates have been observed in people with obesity [44–46] and it may be posited that this would diminish taste responses. However, other evidence indicates no difference in flow rates in people with and without obesity [47,48]. Both acute [17] and habitual [14] fat intake reportedly downregulate expression of NEFA receptors, which could reduce sensitivity, but evidence is based only on very preliminary data and, to be relevant, requires the unsubstantiated assumption that people with obesity necessarily select higher fat diets [21,49]. Indeed, work reporting associations between fat intake and taste sensitivity demonstrate that fat intake did not differ between lean and obese participants at baseline assessments [14]. Finally, even if sensitivity or intensity ratings differed in people with or without obesity, this would not necessarily translate into altered fat taste preferences, intake or body adiposity as taste is only one of myriad influences on food choice and consumption. Taken together, there are hypotheses but no compelling mechanistic evidence that people with obesity should or do differ in fat taste sensitivity or responsiveness from their lean counterparts. This is borne out by the present meta-analysis which revealed no difference in threshold sensitivity or supra-threshold intensity ratings among individuals who are lean, overweight or obese.

Still, the observation that published studies only report no difference or diminished oleogustus in people with obesity warrants consideration. There are no reports of heightened sensitivity or intensity ratings by this group as might be expected if findings are truly random and there was no systematic difference. There are no clear data to account for this, but several testable hypotheses may be proposed. It is possible that people with obesity simply do not perform as well on the types of sensory tests that have been used to measure oleogustus [16]. Given the fact that NEFA impart unpleasant sensations (albeit ones that may become desirable with repeated exposure in given contexts), there could be publication bias as it would be necessary to assume decreased taste responsiveness to account for greater intake. Additionally, there may be confounding factors that contribute to lower observed sensitivity in people with obesity such as higher medication use and/or associated health disorders that alter taste function that were inadvertently selected for in trials reporting differential effects.

### Limitations

As with any meta-analysis, conclusions are influenced by the studies selected for inclusion. There are, at present, a limited number of studies from which data can be obtained. The majority of the studies included come from two main research groups that differ on the composition of the test stimuli. These differences may account for the variability in findings across the studies.

During the data analysis portion of this study, another study concluded that there was a positive association between fatty acid taste sensitivity and BMI [50]. This study was not included in the analysis as there were only 2 overweight and 0 obese individuals tested. We also excluded animal studies from this analysis. While there is a great deal of rodent literature on oleogustus, and these studies are important for understanding underlying mechanisms, the fact that rodents find NEFA attractive rather than aversive [51] suggests significant species differences directly relevant to the question of oleogustus and BMI. Regarding the use of VAS to measure intensity, there is debate as to whether this tool is appropriate to use in taste studies. VAS are easy to understand and use, and unless there is a basis to expect the two groups have different experiences with the stimulus, it is not clear that comparisons would be invalid. However, should there be an underlying difference in the sensory experience of NEFA between the groups, the validity of a VAS response scale may be uncertain. Finally, we chose to limit our investigation to studies that relied upon model stimuli to be able to assess the role of taste in the detection of NEFA. These studies may fail to assess real world conditions and experiences.

### Suggestions for Future Research

We chose to examine studies that used simple stimuli; these model systems attempt to mask textural and olfactory cues to isolate the role of taste. Textural and olfactory cues are important to fat detection [52], are present when eating in naturalistic conditions, and integration of these modalities differs between lean and obese women [53]. Future work should explore the integration of gustation, olfaction, and somatosensation to the sensory perception and appeal of fat and fatty acids in standardized and well-characterized food matrices.

## Conclusions

The present meta-analysis reveals no significant association between oleogustus and BMI. The literature is currently comprised of a limited number of controlled studies with very small samples and one large trial conducted in a non-laboratory setting. Consequently, the basis for drawing firm conclusions is weak. Until well-controlled, larger trials are conducted to establish or challenge the validity of the current findings, it would be prudent to withhold speculation about a causal relationship between weight status and NEFA taste sensitivity.

## Supporting Information

S1 PRISMA Checklist PRISMA Checklist. (DOCX)

S1 Table Excluded studies based on full-text reading. *Taste sensitivity was determined by assessing whether or not the participant could detect NEFA at a specific concentration.
(DOCX)
