# Supplementary material for: Comparisons of Fatty Acid Taste Detection Thresholds in People Who Are Lean vs. Overweight or Obese: A Systematic Review and Meta-Analysis
Source: PLoS One. 2017 Jan 6;12(1):e0169583. doi: 10.1371/journal.pone.0169583 (PMC5218398; doi:10.1371/journal.pone.0169583)
Supplement: S1 Table — *Taste sensitivity was determined by assessing whether or not the participant could detect NEFA at a specific concentration. (DOCX) [file pone.0169583.s002.docx]

**Table S1. Excluded studies based on full-text reading**

| Authors | Reason(s) for exclusion |
| --- | --- |
| Armstrong, 2008 [1] | No adiposity or BMI status of participants provided. |
| Baur, Grosch, Wieser and Jugel, 1977 [2] | No adiposity or BMI status of participants provided; stimuli were oxidized. |
| Chalé-Rush, Burgess and Mattes, 2007 [3] | No adiposity or BMI status of participants provided. |
| Coulon, Miller, Reed and Martin, 2012 [4] | Utilized triglycerides or foods with uncharacterized NEFA profiles as taste stimuli. |
| Crystal, Frye and Kanarek, 1995 [5] | Utilized triglycerides or foods with uncharacterized NEFA profiles as taste stimuli. |
| Drewnowski, 1984 [6] | Utilized triglycerides or foods with uncharacterized NEFA profiles as taste stimuli. |
| Drewnowski, 1990 [7] | Utilized triglycerides or foods with uncharacterized NEFA profiles as taste stimuli. |
| Drewnowski and Schwartz, 1990 [8] | Utilized triglycerides or foods with uncharacterized NEFA profiles as taste stimuli. |
| Drewnowski, Kurth and Rahaim, 1991 [9] | Utilized triglycerides or foods with uncharacterized NEFA profiles as taste stimuli. |
| Drewnowski, Shrager, Lipsky, Stellar and Greenwood, 1989 [10] | Utilized triglycerides or foods with uncharacterized NEFA profiles as taste stimuli. |
| Haryono, Sprajcer and Keast, 2014 [11] | No taste sensitivity measures. |
| Keast, Azzopardi, Newman and Haryono, 2014 [12] | No taste sensitivity measures.* |
| Kulkarni and Mattes, 2013 [13] | No comparison of results by BMI classification provided. |
| Kulkarni and Mattes, 2014 [14] | No taste sensitivity measures. |
| Liang, Sakimura, May, Breen, Driggin, Tepper, Chung and Keller, 2012 [15] | Utilized triglycerides or foods with uncharacterized NEFA profiles as taste stimuli. |
| Martinez-Ruiz, Lopez-Diaz, Wall-Medrano, Jimenez-Castro and Angulo, 2014 [16] | Use of nose clips not specified. |
| Mattes, 2007 [17] | No taste sensitivity measures for LA. |
| Mattes, 2009 [18] | No comparison of results by BMI classification provided. |
| Mela, Langley and Martin, 1994 [19] | Utilized triglycerides or foods with uncharacterized NEFA profiles as taste stimuli. |
| Melis, Sollai, Muroni, Crnjar and Barbarossa, 2015 [20] | No comparison of results by BMI classification provided (BMI < 25.3 kg/m^2^ for all). |
| Mounayar, Morzel, Brignot, Tremblay-Franco, Canlet, Lucchi, Ducoroy, Feron and Neyraud, 2014 [21] | No adiposity or BMI status of participants provided. |
| Mounayar, Septier, Chabanet, Feron and Neyraud, 2013 [22] | No taste sensitivity measures.* |
| Mrizak, Sery, Plesnik, Arfa, Fekih, Bouslema, Zaouali, Tabka and Khan, 2015 [23] | No comparison of results by BMI classification provided (all obese). |
| Nachtsheim and Schlich, 2013 [24] | Utilized triglycerides or foods with uncharacterized NEFA profiles as taste stimuli. |
| Newman and Keast, 2013 [25] | No comparison of results by BMI classification provided (overweight/obese N=2). |
| Pepino and Mennella, 2014 [26] | Utilized triglycerides or foods with uncharacterized NEFA profiles as taste stimuli; medical history (smokers). |
| Pepino, Love-Gregory, Klein and Abumrad, 2012 [27] | No comparison of results by BMI classification provided (all obese). |
| Poette, Mekoué, Neyraud, Berdeaux, Renault, Guichard, Genot and Feron, 2014 [28] | No adiposity or BMI status of participants provided. |
| Running and Mattes, 2014 [29] | No comparison of results by BMI classification provided (overweight/obese N=3). |
| Running and Mattes, 2015 [30] | No adiposity or BMI status of participants provided (no summary information). |
| Schebendach, Klein, Mayer, Devlin, Attia and Walsh, 2014 [31] | Utilized triglycerides or foods with uncharacterized NEFA profiles as taste stimuli. |
| Wajid and Halpern, 2012 [32] | No taste sensitivity measures. |

*Taste sensitivity was determined by assessing whether or not the participant could detect NEFA at a specific concentration.

References

1. Armstrong CLH. 6-n-propylthiouracil: orosensory influence on taste, diet, and chronic disease risk. Purdue University. 2007.

2. Baur C, Grosch W, Wieser H, Jugel H. Enzymatic oxydation of linoleic acid: formation of bittertasting fatty acids. Z Für Lebensm-Unters Forsch. 1977;164: 171–176.

3. Chale-Rush A, Burgess JR, Mattes RD. Multiple routes of chemosensitivity to free fatty acids in humans. Am J Physiol Gastrointest Liver Physiol. 2007;292: G1206–G1212.

4. Coulon SM, Miller AC, Reed JM, Martin CK. Reliability of a common solution-based taste perception test: implications for validity and a briefer test. Eat Behav. 2012;13: 42–45.

5. Crystal S, Frye CA, Kanarek RB. Taste preferences and sensory perceptions in female varsity swimmers. Appetite. 1995;24: 25–36.

6. Drewnowski A. New techniques: multidimensional analyses of taste responsiveness. Int J Obes. 1984;8: 599–607.

7. Drewnowski A. Dietary fats: perceptions and preferences. J Am Coll Nutr. 1990;9: 431–435.

8. Drewnowski A, Schwartz M. Invisible fats: sensory assessment of sugar/fat mixtures. Appetite. 1990;14: 203–217.

9. Drewnowski A, Kurth CL, Rahaim JE. Taste preferences in human obesity: environmental and familial factors. Am J Clin Nutr. 1991;54: 635–641.

10. Drewnowski A, Shrager EE, Lipsky C, Stellar E, Greenwood MRC. Sugar and fat: sensory and hedonic evaluation of liquid and solid foods. Physiol Behav. 1989;45: 177–183.

11. Haryono RY, Sprajcer MA, Keast RS. Measuring oral fatty acid thresholds, fat perception, fatty food liking, and papillae density in humans. J Vis Exp JoVE. 2014;(88). doi: 10.3791/51236. doi:10.3791/51236.

12. Keast RSJ, Azzopardi KM, Newman LP, Haryono RY. Impaired oral fatty acid chemoreception is associated with acute excess energy consumption. Appetite. 2014;80: 1–6.

13. Kulkarni B, Mattes RD. Evidence for Presence of Nonesterified Fatty Acids as Potential Gustatory Signaling Molecules in Humans. Chem Senses. 2013;38: 119–127.

14. Kulkarni BV, Mattes RD. Lingual lipase activity in the orosensory detection of fat by humans. Am J Physiol-Regul Integr Comp Physiol. 2014;306: R879–R885. doi:10.1152/ajpregu.00352.2013.

15. Liang LC, Sakimura J, May D, Breen C, Driggin E, Tepper BJ, et al. Fat discrimination: a phenotype with potential implications for studying fat intake behaviors and obesity. Physiol Behav. 2012;105: 470–475.

16. Martinez-Ruiz NR, Lopez-Diaz J, Wall-Medrano A, Jimenez-Castro JA, Angulo O. Oral fat perception is related with body mass index, preference and consumption of high-fat foods. Physiol Behav. 2014;129: 36–42.

17. Mattes RD. Effects of linoleic acid on sweet, sour, salty, and bitter taste thresholds and intensity ratings of adults. Am J Physiol - Gastrointest Liver Physiol. 2007;292: G1243–G1248.

18. Mattes RD. Oral Detection of Short-, Medium-, and Long-Chain Free Fatty Acids in Humans. Chem Senses. 2009;34: 145–150.

19. Mela DJ, Langley KR, Martin A. No effect of oral or sample temperature on sensory assessment of fat content. Physiol Behav. 1994;56: 655–658.

20. Melis M, Sollai G, Muroni P, Crnjar R, Barbarossa IT. Associations between Orosensory Perception of Oleic Acid, the Common Single Nucleotide Polymorphisms (rs1761667 and rs1527483) in the CD36 Gene, and 6-n-Propylthiouracil (PROP) Tasting. Nutrients. 2015;7: 2068–2084.

21. Mounayar R, Morzel M, Brignot H, Tremblay-Franco M, Canlet C, Lucchi G, et al. Nutri-metabolomics Applied to Taste Perception Phenotype: Human Subjects with High and Low Sensitivity to Taste of Fat Differ in Salivary Response to Oleic Acid. Omics J Integr Biol. 2014;18: 666–672.

22. Mounayar R, Septier C, Chabanet C, Feron G, Neyraud E. Oral Fat Sensitivity in Humans: Links to Saliva Composition Before and After Stimulation by Oleic Acid. Chemosens Percept. 2013;6: 118–126.

23. Mrizak I, Šerý O, Plesnik J, Arfa A, Fekih M, Bouslema A, et al. The A allele of cluster of differentiation 36 (CD36) SNP 1761667 associates with decreased lipid taste perception in obese Tunisian women. Br J Nutr. 2015;113: 1330–1337. doi:10.1017/S0007114515000343.

24. Nachtsheim R, Schlich E. The influence of 6-n-propylthiouracil bitterness, fungiform papilla count and saliva flow on the perception of pressure and fat. Food Qual Prefer. 2013;29: 137–145.

25. Newman LP, Keast RS. The test–retest reliability of fatty acid taste thresholds. Chemosens Percept. 2013;6: 70–77.

26. Pepino MY, Mennella JA. Cigarette smoking and obesity are associated with decreased fat perception in women. Obesity. 2014;22: 1050–1055.

27. Pepino MY, Love-Gregory L, Klein S, Abumrad NA. The fatty acid translocase gene, CD36, and lingual lipase influence oral sensitivity to fat in obese subjects. J Lipid Res. 2012;53: 561–566.

28. Poette J, Mekoué J, Neyraud E, Berdeaux O, Renault A, Guichard E, et al. Fat sensitivity in humans: oleic acid detection threshold is linked to saliva composition and oral volume. Flavour Fragr J. 2014;29: 39–49.

29. Running CA, Mattes RD. Different oral sensitivities to and sensations of short-, medium-, and long-chain fatty acids in humans. Am J Physiol Gastrointest Liver Physiol. 2014;307: G–381–G389.

30. Running CA, Mattes RD. Humans are more sensitive to the taste of linoleic and alpha-linolenic than oleic acid. Am J Physiol Liver Physiol. 2015;308: G442–9. doi:10.1152/ajpgi.00394.2014

31. Schebendach JE, Klein DA, Mayer LE, Devlin MJ, Attia E, Walsh BT. Assessment of fat taste in individuals with and without anorexia nervosa. Int J Eat Disord. 2014;47: 215–218.

32. Wajid NA, Halpern BP. Oral cavity discrimination of vapor-phase long-chain 18-carbon fatty acids. Chem Senses. 2012;37: 595–602.
